# Supplementary material for: Pyruvate Dehydrogenase Kinase 1 inhibition mediated oxidative phosphorylation enhancement in cartilage promotes osteoarthritis progression
Source: BMC Musculoskelet Disord. 2023 Jul 20;24:597. doi: 10.1186/s12891-023-06585-6 (PMC10357736; doi:10.1186/s12891-023-06585-6)
Supplement: Supplementary file 1 — Additional file 1. [file 12891_2023_6585_MOESM1_ESM.zip › MF.Gsea.1653627316242/GOMF_INTRAMOLECULAR_OXIDOREDUCTASE_ACTIVITY.html]

Details for gene set GOMF\_INTRAMOLECULAR\_OXIDOREDUCTASE\_ACTIVITY[GSEA]

|  || Dataset | 55457\_collapsed\_to\_symbols.cls.cls #OA\_versus\_normal.cls.cls #OA\_versus\_normal\_repos |
| Phenotype | cls.cls#OA\_versus\_normal\_repos |
| Upregulated in class | OA |
| GeneSet | GOMF\_INTRAMOLECULAR\_OXIDOREDUCTASE\_ACTIVITY |
| Enrichment Score (ES) | 0.5145858 |
| Normalized Enrichment Score (NES) | 2.0117536 |
| Nominal p-value | 0.0 |
| FDR q-value | 0.007690799 |
| FWER p-Value | 0.092 |
Table: GSEA Results Summary

  

Fig 1: Enrichment plot: GOMF\_INTRAMOLECULAR\_OXIDOREDUCTASE\_ACTIVITY      
 Profile of the Running ES Score & Positions of GeneSet Members on the Rank Ordered List

  

| SYMBOL | TITLE | RANK IN GENE LIST | RANK METRIC SCORE | RUNNING ES | CORE ENRICHMENT || 1 | CRELD2 | cysteine rich with EGF like domains 2 [Source:HGNC Symbol;Acc:HGNC:28150] | 146 | 0.867 | 0.0508 | Yes |
| 2 | GPI | glucose-6-phosphate isomerase [Source:HGNC Symbol;Acc:HGNC:4458] | 166 | 0.842 | 0.1096 | Yes |
| 3 | MPI | mannose phosphate isomerase [Source:HGNC Symbol;Acc:HGNC:7216] | 255 | 0.781 | 0.1587 | Yes |
| 4 | PDIA5 | protein disulfide isomerase family A member 5 [Source:HGNC Symbol;Acc:HGNC:24811] | 324 | 0.735 | 0.2061 | Yes |
| 5 | MIF | macrophage migration inhibitory factor [Source:HGNC Symbol;Acc:HGNC:7097] | 555 | 0.640 | 0.2340 | Yes |
| 6 | ENOX1 | ecto-NOX disulfide-thiol exchanger 1 [Source:HGNC Symbol;Acc:HGNC:25474] | 558 | 0.638 | 0.2796 | Yes |
| 7 | HPGDS | hematopoietic prostaglandin D synthase [Source:HGNC Symbol;Acc:HGNC:17890] | 605 | 0.622 | 0.3205 | Yes |
| 8 | ECI2 | enoyl-CoA delta isomerase 2 [Source:HGNC Symbol;Acc:HGNC:14601] | 669 | 0.601 | 0.3587 | Yes |
| 9 | ERP29 | endoplasmic reticulum protein 29 [Source:HGNC Symbol;Acc:HGNC:13799] | 706 | 0.592 | 0.3983 | Yes |
| 10 | P4HB | prolyl 4-hydroxylase subunit beta [Source:HGNC Symbol;Acc:HGNC:8548] | 777 | 0.575 | 0.4340 | Yes |
| 11 | DDT | D-dopachrome tautomerase [Source:HGNC Symbol;Acc:HGNC:2732] | 1094 | 0.498 | 0.4451 | Yes |
| 12 | ERP44 | endoplasmic reticulum protein 44 [Source:HGNC Symbol;Acc:HGNC:18311] | 1175 | 0.481 | 0.4733 | Yes |
| 13 | ECI1 | enoyl-CoA delta isomerase 1 [Source:HGNC Symbol;Acc:HGNC:2703] | 1453 | 0.435 | 0.4830 | Yes |
| 14 | GNPDA1 | glucosamine-6-phosphate deaminase 1 [Source:HGNC Symbol;Acc:HGNC:4417] | 1795 | 0.385 | 0.4840 | Yes |
| 15 | ECH1 | enoyl-CoA hydratase 1 [Source:HGNC Symbol;Acc:HGNC:3149] | 1901 | 0.373 | 0.5026 | Yes |
| 16 | PDIA6 | protein disulfide isomerase family A member 6 [Source:HGNC Symbol;Acc:HGNC:30168] | 2073 | 0.353 | 0.5146 | Yes |
| 17 | PTGIS | prostaglandin I2 synthase [Source:HGNC Symbol;Acc:HGNC:9603] | 2654 | 0.284 | 0.4899 | No |
| 18 | PIGK | phosphatidylinositol glycan anchor biosynthesis class K [Source:HGNC Symbol;Acc:HGNC:8965] | 2801 | 0.270 | 0.4978 | No |
| 19 | TXNDC5 | thioredoxin domain containing 5 [Source:HGNC Symbol;Acc:HGNC:21073] | 3378 | 0.214 | 0.4683 | No |
| 20 | HSD3B1 | "hydroxy-delta-5-steroid dehydrogenase, 3 beta- and steroid delta-isomerase 1 [Source:HGNC Symbol;Acc:HGNC:5217]" | 3695 | 0.186 | 0.4571 | No |
| 21 | PDIA4 | protein disulfide isomerase family A member 4 [Source:HGNC Symbol;Acc:HGNC:30167] | 3756 | 0.181 | 0.4654 | No |
| 22 | EBP | EBP cholestenol delta-isomerase [Source:HGNC Symbol;Acc:HGNC:3133] | 3848 | 0.171 | 0.4705 | No |
| 23 | TPI1 | triosephosphate isomerase 1 [Source:HGNC Symbol;Acc:HGNC:12009] | 4059 | 0.150 | 0.4649 | No |
| 24 | TBXAS1 | thromboxane A synthase 1 [Source:HGNC Symbol;Acc:HGNC:11609] | 4338 | 0.125 | 0.4523 | No |
| 25 | PTGES | prostaglandin E synthase [Source:HGNC Symbol;Acc:HGNC:9599] | 4399 | 0.121 | 0.4563 | No |
| 26 | HSD3B2 | "hydroxy-delta-5-steroid dehydrogenase, 3 beta- and steroid delta-isomerase 2 [Source:HGNC Symbol;Acc:HGNC:5218]" | 4424 | 0.119 | 0.4630 | No |
| 27 | CRELD1 | cysteine rich with EGF like domains 1 [Source:HGNC Symbol;Acc:HGNC:14630] | 5555 | 0.028 | 0.3771 | No |
| 28 | PTGDS | prostaglandin D2 synthase [Source:HGNC Symbol;Acc:HGNC:9592] | 5583 | 0.025 | 0.3768 | No |
| 29 | ENOPH1 | enolase-phosphatase 1 [Source:HGNC Symbol;Acc:HGNC:24599] | 5615 | 0.023 | 0.3760 | No |
| 30 | HYI | hydroxypyruvate isomerase (putative) [Source:HGNC Symbol;Acc:HGNC:26948] | 5777 | 0.010 | 0.3642 | No |
| 31 | IDI1 | isopentenyl-diphosphate delta isomerase 1 [Source:HGNC Symbol;Acc:HGNC:5387] | 5842 | 0.006 | 0.3597 | No |
| 32 | GLRX2 | glutaredoxin 2 [Source:HGNC Symbol;Acc:HGNC:16065] | 6116 | -0.014 | 0.3394 | No |
| 33 | QSOX1 | quiescin sulfhydryl oxidase 1 [Source:HGNC Symbol;Acc:HGNC:9756] | 6130 | -0.015 | 0.3395 | No |
| 34 | PTGES2 | prostaglandin E synthase 2 [Source:HGNC Symbol;Acc:HGNC:17822] | 6269 | -0.025 | 0.3305 | No |
| 35 | PDIA3 | protein disulfide isomerase family A member 3 [Source:HGNC Symbol;Acc:HGNC:4606] | 6607 | -0.050 | 0.3079 | No |
| 36 | EHHADH | enoyl-CoA hydratase and 3-hydroxyacyl CoA dehydrogenase [Source:HGNC Symbol;Acc:HGNC:3247] | 7968 | -0.153 | 0.2131 | No |
| 37 | PTGES3 | prostaglandin E synthase 3 [Source:HGNC Symbol;Acc:HGNC:16049] | 8802 | -0.217 | 0.1638 | No |
| 38 | PDIA2 | protein disulfide isomerase family A member 2 [Source:HGNC Symbol;Acc:HGNC:14180] | 8961 | -0.229 | 0.1679 | No |
| 39 | RPIA | ribose 5-phosphate isomerase A [Source:HGNC Symbol;Acc:HGNC:10297] | 9793 | -0.301 | 0.1249 | No |
| 40 | SREBF2 | sterol regulatory element binding transcription factor 2 [Source:HGNC Symbol;Acc:HGNC:11290] | 10070 | -0.328 | 0.1269 | No |
| 41 | ITGB3 | integrin subunit beta 3 [Source:HGNC Symbol;Acc:HGNC:6156] | 10310 | -0.352 | 0.1335 | No |
| 42 | DCT | dopachrome tautomerase [Source:HGNC Symbol;Acc:HGNC:2709] | 10995 | -0.429 | 0.1110 | No |
| 43 | GSTA1 | glutathione S-transferase alpha 1 [Source:HGNC Symbol;Acc:HGNC:4626] | 11608 | -0.517 | 0.1005 | No |
Table: GSEA details [plain text format]

  

Fig 2: GOMF\_INTRAMOLECULAR\_OXIDOREDUCTASE\_ACTIVITY      
 Blue-Pink O' Gram in the Space of the Analyzed GeneSet

  

Fig 3: GOMF\_INTRAMOLECULAR\_OXIDOREDUCTASE\_ACTIVITY: Random ES distribution      
 Gene set null distribution of ES for **GOMF\_INTRAMOLECULAR\_OXIDOREDUCTASE\_ACTIVITY**
